# Spatial heterogeneity of soil factors enhances intraspecific variation in plant functional traits in a desert ecosystem

**DOI:** 10.3389/fpls.2024.1504238

**Published:** 2024-12-23

**Authors:** Yong-chang Wang, Xue-ni Zhang, Ji-fen Yang, Jing-ye Tian, Dan-hong Song, Xiao-hui Li, Shuang-fu Zhou

**Affiliations:** ^1^ College of Ecology and Environment, Xinjiang University, Urumqi, China; ^2^ Key Laboratory of Oasis Ecology of Education Ministry, Urumqi, China; ^3^ Xinjiang Jinghe Observation and Research Station of Temperate Desert Ecosystem, Ministry of Education, Urumqi, China

**Keywords:** plant functional traits, plant adaptation strategies, desert plants, soil moisture-salinity habitat, habitat filtering effect

## Abstract

**Introduction:**

Functional traits of desert plants exhibit remarkable responsiveness, adaptability and plasticity to environmental heterogeneity.

**Methods:**

In this study, we measured six crucial plant functional traits (leaf carbon, leaf nitrogen, leaf phosphorus, leaf thickness, chlorophyll concentration, and plant height) and employed exemplar analysis to elucidate the effects of soil environmental heterogeneity on intraspecific traits variation in the high-moisture-salinity and low-moisture-salinity habitats of the Ebinur LakeWetland National Nature Reserve.

**Results:**

The results showed that (1) The soil moisture and electrical conductivity heterogeneity showed significant differences between the two moisture-salinity habitats. Moreover, soil nutrient in high moisture-salinity habitat exhibited higher heterogeneity than in low moisture-salinity habitat. The order of intraspecific trait variation among different life forms was herbs > shrubs > trees in both the soil moisture-salinity habitats. (2) At the community level, intraspecific variation of leaf carbon, nitrogen, plant height and chlorophyll content in high moisture-salinity habitat was higher than that in low moisture-salinity habitat, while the opposite was true for leaf thickness and leaf phosphorus content. (3) Our findings revealed a positive impact of soil heterogeneity on intraspecific traits variation. In high moisture-salinity habitat, the heterogeneity of soil organic carbon had the highest explanatory power for intraspecific traits variation, reaching up to 20.22%, followed by soil total nitrogen (9.55%) and soil total phosphorus (3.49%). By comparison, in low-moisture-salinity habitat, the heterogeneity of soil moisture alone contributes the highest explanatory power for intraspecific traits variation in community-level, reaching up to 13.89%, followed by the heterogeneity of soil total nitrogen (3.76%).

**Discussion:**

This study emphasizes the differences in soil heterogeneity and intraspecific trait variation among plant life forms under various soil moisture-salinity habitats and confirms the significant promoting effect of soil heterogeneity on intraspecific trait variation of desert plant. Our findings provide valuable theoretical basis and reference for predicting plant adaptation strategies under environmental change scenarios.

## 1 Introduction

Intraspecific trait variation (ITV) depends on the available phenotypic trait plasticity of individuals within a population. Phenotypic plasticity, that is, the phenotypic variation expressed by a single genotype under different environmental conditions (Nicotra et al., 2010). ITV contributes significantly to the persistence of plants in variable environments, in which abiotic factors play a non-negligible role (Stanton et al., 2000; Valladares et al., 2007). Soil heterogeneity refers to the uneven distribution of soil resources, such as nutrients and water (Feeser et al., 2018). Soil heterogeneity significantly affects plant growth and species coexistence (Liu et al., 2021; Olagoke et al., 2023), thus it is an important driver of plant community structure and dynamics (Lundholm, 2009). Few previous studies have reported on the important effects of soil heterogeneity within habitats on ITV in plant communities, especially in arid zones with sparse vegetation and environmental extremes.

Ecologists have long used the mean trait values of species to infer ecological processes (McGill et al., 2006). Nonetheless, current researches have demonstrated a significant amount of ITV (Stojnic et al., 2022; Westerband et al., 2021). For example, a recent meta-analysis has provided a comprehensive insight into the matter, indicating that ITV is responsible for 25%–31% of the variation in commonly measured traits within plant communities (Siefert et al., 2015). For example, Hurtado et al. have showed that intraspecific trait variability explained most of the functional changes in lichen communities in response to the latitudinal gradient (Hurtado et al., 2020). These findings emphasize the significant role of ITV on community assembly and ecosystem processes (Hart et al., 2016; Pérez-Izquierdo et al., 2019; Siefert et al., 2015; Turcotte and Levine, 2016). Phenotypic plasticity, which refers to the variation in phenotype expressed by a single genotype under varying environmental conditions (Nicotra et al., 2010; Sultan, 2000), is considered one of the key mechanisms through which plants respond to environmental changes (Arnold et al., 2019; Gratani, 2014). As an illustration, the ITV observed in *Eremanthus erythropappus* in Brazil has been shown to enhance its capability to thrive in various habitats, including forests and savannas (Silva et al., 2019). Likewise, a study on *Brachypodium hybridum*, an annual grass species in California, has revealed that ITV in seed biomass promotes its successful colonization in water-limited environments (Liu et al., 2019). Ecological niche theory suggests that species in an ecosystem occupy different ecological niches and vary with environmental gradients, so their ecological strategies change accordingly (Kearney and Porter, 2006; Violle and Jiang, 2009). It has been proposed that ITV characterizes the maximum fitness of a species and fundamentally determines the ecological niche width of a plant; Jung et al (2010) argued that ITV accounted for 44% of the variation in species’ traits and increased the chances of a species being unfiltered by the environment, thus facilitating the coexistence of species (Jung et al., 2010).

It is generally recognized that heterogeneous environments typically contain more ecological niches than homogeneous environments (Chesson, 2000) and thus drive variations in plant functional traits (Des Roches et al., 2021; Wan et al., 2019), improve plant adaptations and positively affect their growth, reproduction and survival (Karbstein et al., 2020). In this sense, soil environmental heterogeneity within habitats can potentially result in an augmentation of the diversity of functional phenotype, thus enhancing ITV in plant traits (Bloomfield et al., 2018; Stark et al., 2017). For example, it has been found that the increase in ITV in Qinghai-Tibetan alpine meadow communities under different altitude conditions was related to soil heterogeneity (Siefert and Ritchie, 2016). However, the effects of soil heterogeneity on plant ITV in other ecosystems is still poorly understood. Therefore, studying intraspecific traits variation in relation to environmental heterogeneity better reflects the effects of soil heterogeneity within habitats, such as nutrient limitation (Frederiksen et al., 2005; Tautenhahn et al., 2019) or drought and salt stress effects (Tusifujiang et al., 2021). At present, the relationship between ITV and soil environmental heterogeneity across different life forms and communities has not received sufficient attention.

Regional variation in plant functional traits is associated with climatic heterogeneity (Símová et al., 2015). Soil heterogeneity tends to be more important to ITV at the local scale. For example, habitats characterized by a pronounced level of microenvironmental heterogeneity at small spatial scales, such as forest understories and alpine meadows are usually more important for ITV than interspecific trait variation. Furthermore, their contribution to intercommunity trait variation decreases with increasing spatial scale (Benes and Bracken, 2020). At small or local scales, topographic and soil factors play a crucial role in shaping the variations of plant traits (Liu and Ma, 2015). For instance, a study has demonstrated the significance of soil depth and moisture heterogeneity in driving variations in plant functional traits (Price et al., 2017). Soil heterogeneity influences both abiotic and biotic screening processes by altering competitive relationships among plants, suggesting that its impact on plant trait differentiation might surpass the effects of resource availability alone. Theoretically, greater plants trait variation resulting from soil heterogeneity contribute to increased functional diversity (Price et al., 2017). However, empirical investigations focusing on the role of soil environmental heterogeneity in elucidating plants trait variation within localized regional environments are limited, particularly in arid desert ecosystems where soil heterogeneity is more pronounced (Li et al., 2014). The northwest of Jinghe County in Xinjiang, which is characterized by its dry climate within the North Temperate Continental zone, is home to the Ebinur Lake Wetland National Nature Reserve (ELWNNR). This reserve, is situated in a typical temperate arid desert ecosystem, experiences limited rainfall and strong evaporation. The desert plants within this region are vital components of global biodiversity and play a significant role in sustaining regional ecological equilibrium. Previous studies have demonstrated the substantial impact of soil moisture-salinity content on functional traits of the plant communities in this region, particularly noting a closer relationship between interspecific and intraspecific trait variability and soil factors in high soil moisture-salinity habitat (Tusifujiang et al., 2021). Nonetheless, it remains uncertain whether soil heterogeneity in the region increased intraspecific variation in plant traits, and how this effect varies across different soil moisture-salinity habitats. To address this uncertainty, we conducted a field investigation and measured the morphological, chemometric and physiological traits of plants and soil heterogeneity in the ELWNNR, and tried to answer the following questions: (1) What’s the differences of ITV among life forms and between different soil moisture-salinity habitats? (2) Does soil heterogeneity promote ITV, and does change of the soil moisture-salinity habitats affect the role of soil heterogeneity on ITV? This study aims to provide novel insights into the response of ITV and plant adaptation to environmental heterogeneity in drylands. Furthermore, it will serve as a valuable reference for the preservation and restoration of desert ecosystem.

## 2 Materials and methods

### 2.1 Overview of the study zone

The study area, ELWNNR is situated in the southwest of the Junggar Basin, Xinjiang. It is the lowest depression and moisture-salinity accumulation center in the southwest margin of the Junggar Basin. Its coordinates are 44°30’ N-45°09’ N and 82°36’ E-83°50’ E, encompassing an zone with a dry environment characterized by uneven intra-annual precipitation distribution, averaging 105.17 mm annually. The region also experiences a high average annual evaporation of 1,315 mm and an average annual temperature of 5°C. The Aqikesu River, a crucial water source for ELWNNR situated on its eastern side, has been significantly impacted by human activity and climate change, leading to the river’s near-complete drying, posing a threat to the degradation of riparian vegetation (**Supplementary Figure S1**). Previous research has highlighted the variation in plant species as well as soil moisture and salinity along the transect vertical to the Aqikesu River, indicating changes in these factors with distance from the river (Zhang et al., 2016). In this study, we focused on intraspecific traits variation of the following species, which are found in both the soil moisture-salinity habitats: the tree specie *Populus euphratica*, the shrubby plants *Nitraria tangutorum*, *Apocynum venetum*, *Alhagi sparsifolia* and *Halimodendron halodendron*, as well as the herbaceous plants *Phragmites australis* and *Karelinia capsica* (Wang et al., 2017) (**Supplementary Table S1**). These species are selected because they are distributed across both soil moisture-salinity habitats, and their population sizes are sufficient to meet the requirements of the study.

### 2.2 Sample plot setting and sampling

To minimize the influence of seasonal plant renewal and metabolic variations, our survey and sampling activities were strategically scheduled to coincide with the peak of plant biomass (July–August). The sample strips and plots were specifically laid out as follows: three sample strips, spaced 1 km apart and running perpendicular to the river channel, were established. Each sample strip extended northward and was investigated at intervals of 0.1 km, resulting in a total of 10 sample sites per strip, covering approximately 1 km in length. At each sample site, three 10 m × 10 m plots were investigated, with a spacing of about 100 m between plots (**Supplementary Figure S1**). This method resulted in 30 sample sites across the three sample strips, totaling 90 sample plots (30×3) were investigated. In this study, GPS was utilized to record the latitude and longitude of the center of each sample plot. Additionally, the species, plant height, and frequency of occurrence within each sample plot were documented. In each sample plot, three healthy, undamaged, and similarly-sized mature leaves were collected from three individuals of the same species (with a frequency greater than 3%). The relative chlorophyll content of these leaves was measured using a SPAD-502 chlorophyll meter. The thickness of each leaf was measured with a vernier caliper, taking care to avoid the main leaf veins and focusing on the central area, with an accuracy of 0.01mm. After leaf thickness measurement, approximately 20 grams of leaf samples were collected from each individual and stored in envelopes for transportation back to the laboratory. There, the leaf samples were analyzed to determine their carbon (C), nitrogen (N), and phosphorus (P) content. To comprehensively reflect the conditions of soil moisture, salinity and nutrients within the sample plots, three soil samples were randomly and uniformly selected within each plot. After removing the litter layer, soil samples at 0-20 cm depth were collected, rapidly mixed, and then bagged as composite samples. In total, 90 soil samples were collected from all sample plots. All soil samples were brought back to the Key Laboratory of Oasis Ecology of Education Ministry in Xinjiang University for further analysis.

### 2.3 Measurement and experimental analysis

Six functional traits were selected for analysis, encompassing morphological traits (leaf thickness, plant height), chemical traits (leaf carbon C, nitrogen N, phosphorus P), and a physiological trait (chlorophyll). Leaf thickness impacts the rate of photosynthesis; as leaf thickness increases, so does the resistance to water diffusion from the leaf interior to the surface, which helps to prevent water evaporation from plant (Liu., 2018). Plant height constitutes an adaptive response to environmental changes, and generally correlated with a plant’s competitive ability. In environments where resources are limited, taller plants may have easier access to essential resources, thereby gaining a competitive edge over other species. The chemical traits, leaf C, N, and P, are essential nutrients that underpin plant photosynthesis, especially leaf N and P concentrations are the most important leaf traits regulating physiological effects and plant growth (Wright et al., 2004). Chlorophyll is a pivotal indicator that influence plant nitrogen profile, photosynthetic capacity and plant growth characteristics (Liu., 2018).

All leaf samples were dried in an oven at 75°C for 48 hours. After drying, the leaves were crushed and sieved to facilitate the determination of leaf C, N, and P content. The specific indices and experimental methods are as follows: the potassium dichromate-sulfate oxidation method was employed to determine the carbon content of leaves. The Kjeldahl method, with H_2_SO_4_ accelerated digestion, was utilized to determined leaf nitrogen content. Total phosphorus in leaves was measured using the H_2_SO_4_-H_2_O_2_ digestion followed by the molybdenum antimony resistance colorimetric method. Soil volumetric water content (SWVC) and electrical conductivity (EC, used to represent for soil salinity) were assessed using the drying method and conductometer (DDS-307, Shanghai INESA Scientific Instrument Co., Ltd., Shanghai, China), respectively. Soil organic carbon (SOC) was determined by the potassium dichromate sulfuric acid oxidation. Soil total nitrogen (TN) was determined by the Kjeldahl. Soil total phosphorus (TP) was measured using the HClO_4_-H_2_SO_4_ molybdenum antimony colorimetric method. All measurement procedures were based on the methods described in (Dong., 1997).

### 2.4 Statistical analysis

#### 2.4.1 High and low soil moisture-salinity habitats delineation

Based on the soil water content and electrical conductivity data of each sample site(n=30), the 90 sample plots were categorized into two groups using cluster analysis (Hierarchical Clustering, euclidean distance) with the “average” method: high soil moisture-salinity habitat(n=36), where soil electrical conductivity ranged from 7.26 to 14.06 mS/cm with a mean value of 10.53 mS/cm, and SWVC ranged from 6.37% to 11.03% with a mean value of 9.28%; low soil moisture-salinity habitat(n=54), where soil electrical conductivity ranged from 3.14 to 4.93 mS/cm with a mean value of 4.52 mS/cm, and SWVC ranged from 1.67% to 5.54% with a mean value of 4.74%. Differences between the two soil moisture-salinity habitats were highly significant (*P < 0.01*).

#### 2.4.2 Soil Heterogeneity and Plant intraspecific trait variation

(1) We employed the coefficient of variation (CV) to measure soil heterogeneity (SH) of each sample site. The formula is:


(1) $$\text{CV}=\frac{\text{SD}}{\text{mean}}\times 100$$

Where SD and mean is the standard deviation and average of soil factor in the three plots from the sample site. We calculated CV for each soil factor and for all soil factors (i.e. SH), where the SH is the mean values of CV for soil water content, soil electrical conductivity, soil organic carbon, soil total nitrogen and soil total phosphorus (Helm et al., 2019).

(2) The coefficient of variation (*CV_1_*) was utilized to characterize the degree of intraspecific traits variation at species level. Intraspecific functional trait variation (iFD_CV_) at species level is the average of *CV_1_* in all traits of the species under the same habitat. The iFD_CV_ of life-forms was calculated as the product of the average of *CV_1_* of six traits of each species in a certain life form and the relative abundance of the species in the life form.

(3) ITV of life-form types is calculated by summing the products of the relative abundance and *CV_1_* of species, which belong to the same life-form type in each sample site.

(4) ITV at community level (CWV) was accessed by referencing the formula of community weighted mean trait (CWM). Specifically, CWV was calculated as the following formula:


(2) $$\text{CWV}=\sum_{\text{i}=1}^{\text{S}}{\text{P}}_{\text{i}}\cdot \text{C}{\text{V}}_{\text{i}}$$

Where $P_{i}\;$ is the relative abundance of species *i* in a given plot, *CV_i_* is the coefficient of variation of species *i* in the plot, and S is the number of species in the plot. Sample site was calculated by taking the average of the plot.

#### 2.4.3 Impact of soil heterogeneity on intraspecific trait variation

Detrended correspondence analysis (DCA) revealed that the gradient lengths of community functional traits across the two soil moisture-salinity habitats were less than 3. Consequently, based on redundancy analysis (RDA), exemplar analysis was conducted to ascertain the relative importance and quantify the individual impact of the heterogeneity of various soil factor on intraspecific traits variation (Lai et al., 2022). We employed the adjusted coefficient of determination ( ${\text{R}}_{\text{adj}}^{2}$ ) instead of the conventional coefficient of determination (*R^2^*) to determine the final analytical results. This method helps to prevent model overfitting in *R^2^* calculations, as discussed by Crawley (Crawley, 2007).

Data were organized and compiled using Excel vers. 2016, and one-way ANOVA was performed with SPSS Amos version 26. Plots were predominantly created with Origin vers. 2025, exemplar analysis and cluster analysis were carried out using R’s ade4, UpSet VP, rdacca.hp and stats packages.

## 3 Results

### 3.1 Soil heterogeneity in different soil moisture-salinity habitats

**Figure 1:**
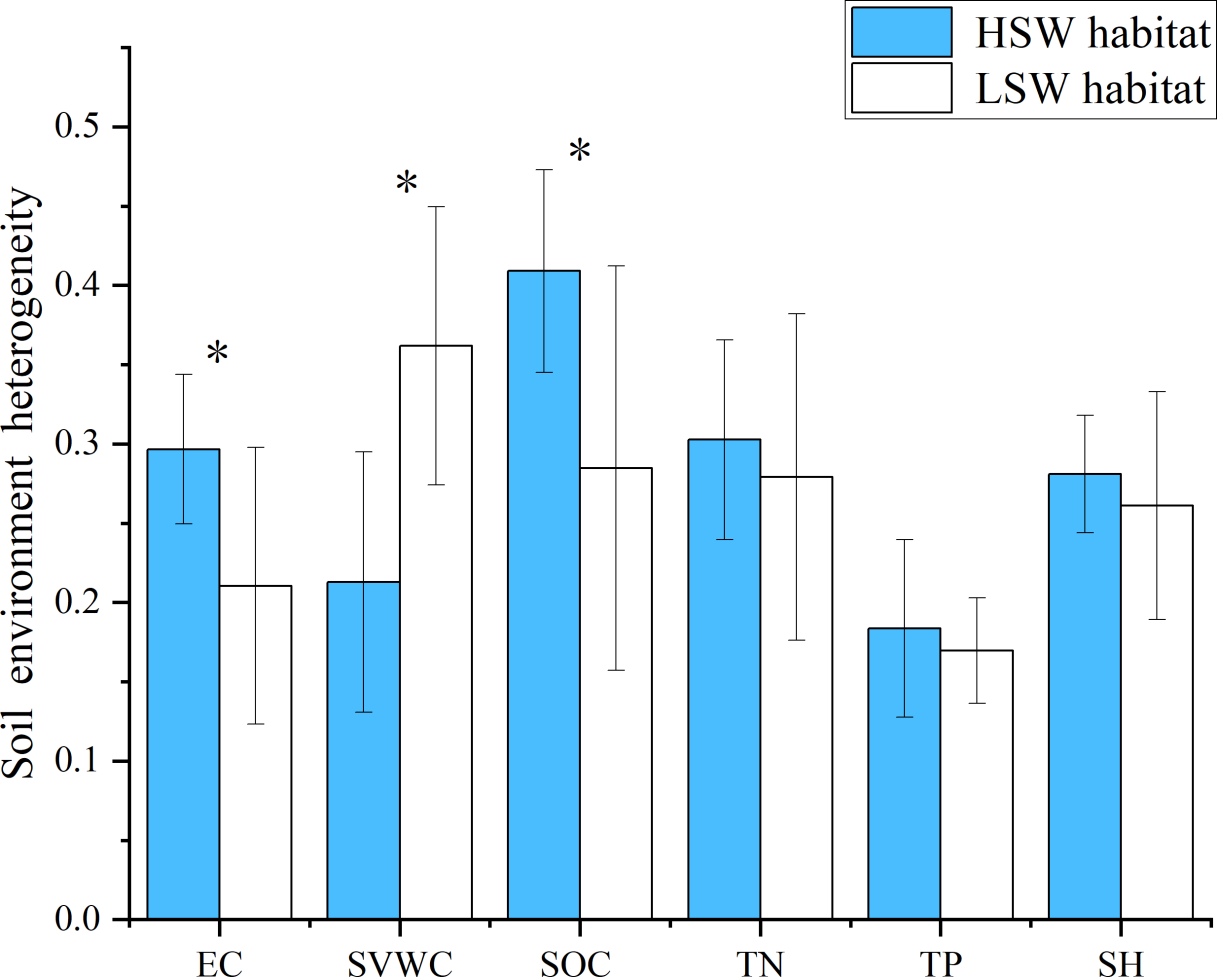
Blue and white represent the two distinct habitats, HSW (High Soil Moisture and Salinity) and LSW (Low Soil Moisture and Salinity), respectively. Each habitat exhibits differences in soil characteristics such as Soil volumetric water content (SVWC), electrical conductivity (EC), Soil organic carbon (SOC), total nitrogen (TN), total phosphorus (TP) and soil heterogeneity (SH). Bar heights indicate the average coefficient of variation (CV) for each soil characteristic. Error bars represent the standard error of the mean (SEM). Asterisks denote significant differences between the two habitats (P < 0.05).

Soil heterogeneity of HSW habitat was generally higher than that of LSW habitat. Specifically, the soil electrical conductivity and organic carbon heterogeneity in HSW habitat were significantly higher than those in LSW habitat (*p<*0.05). The soil TN, TP heterogeneity and SH in HSW habitat were slightly higher than those in LSW habitat (*p>*0.05). However, the soil SWVC heterogeneity in HSW habitat was significantly lower than that in LSW habitat (*p<*0.05). The SOC heterogeneity was the highest in HSW habitat, the soil SWVC heterogeneity was the highest in LSW habitat, and the soil TP heterogeneity was the lowest in both habitats (**Figure 1**).

### 3.2 Plant intraspecific trait variation across soil moisture-salinity habitats

Among the six plant functional traits of herb, shrub and tree plants, the ITV of leaf carbon (C) is the lowest in both the habitats, which ranging from 3.85% to 6.14%. In contrast, plant height was the highest ITV in the two habitats, with a range of 20.40% to 27.52% (**Table 1**). The high moisture-salinity habitat exhibited greater ITV in leaf nitrogen (N), plant height, and chlorophyll among herbs, shrubs, and trees. When considering the ITV of the six functional traits, the order was herb > shrub > tree, but the ITV of leaf thickness (LT) and leaf phosphorus (P) are lower in HSW habitat compared to LSW habitat. Among different life forms, herbaceous plants demonstrated the highest ITV for functional traits other than chlorophyll.

**Table 1:** Changes in ITV of life-forms and community traits in two soil moisture and salinity habitats (mean ± standard deviation).

Plant traits	High Soil Moisture and Salinity habitat				Low Soil Moisture and Salinity habitat			
	Tree	Shrub	Herb	Community	Tree	Shrub	Herb	Community
**Leaf carbon**	3.85 ± 1.75	4.50 ± 1.04	6.14 ± 1.78	5.26 ± 1.58	4.69 ± 2.62	4.72 ± 1.75	5.45 ± 2.11	5.03 ± 0.99
**Leaf nitrogen**	15.58 ± 11.50	15.69 ± 5.30	21.15 ± 8.73	18.48 ± 4.79	10.09 ± 7.54	12.45 ± 5.59	17.80 ± 8.82	17.30 ± 5.03
**Leaf phosphorus**	6.56 ± 3.78	13.62 ± 2.86	17.76 ± 9.22	15.91 ± 5.13	11.57 ± 4.54	15.25 ± 4.66	21.61 ± 10.44	23.24 ± 11.37
**Leaf thickness**	11.50 ± 5.12	11.74 ± 4.59	17.07± 4.46	15.84 ± 4.79	13.09 ± 5.58	12.06 ± 4.83	18.62 ± 8.92	16.06 ± 8.66
**Chlorophyll**	8.30 ± 3.80	10.96 ± 2.64	13.58 ± 3.20	10.83 ± 2.21	7.11 ± 5.01	13.55 ± 2.87	10.58 ± 3.15	11.12 ± 3.97
**Plant height**	24.42 ± 10.28	25.33 ± 9.65	27.52 ± 10.69	29.41 ± 9.48	20.40 ± 2.99	21.05 ± 8.67	23.87 ± 9.22	26.77 ± 8.37
$\text{iF}{\text{D}}_{\text{CV}}$	10.79 ± 2.83	14.79 ± 2.42	16.56 ± 3.91	15.84 ± 2.37	12.07 ± 1.51	13.47 ± 2.82	16.47 ± 3.79	15.06 ± 3.42

The bolded text indicate plant functional traits and index that were calculated for trait variation in this study.

In the HSW habitat, ITV among different life forms generally followed the order of herbs > shrubs > trees, with the exception of chlorophyll, showing a pattern of shrubs > herbs > trees. However, in the LSW habitat, ITV of all traits among life forms consistently followed the order of herbs > shrubs > trees.

At the community level, iFD_CV_ and ITV of C, N, LT, and CH did not vary significantly between the two habitats. The ITV of C, N, and H in the HSW habitat were slightly lower than those in the LSW habitat, while the ITV of LT and CH in the LSW habitat were slightly higher than those in the HSW habitat. Among all the traits at the community level, the ITV of C was the lowest under both habitats, but the higher ITV were H and P in the HSW habitat, and H and N in the LSW habitat.

### 3.3 Relationship between plant intraspecific trait variation and soil heterogeneity

There were significant positive correlations between plant ITV and the heterogeneity of various soil environmental factors (**Supplementary Figure S2**). Positive correlations were found between leaf N and leaf thickness variability and soil moisture heterogeneity, and between leaf N variability and soil salinity heterogeneity. Some other traits were positively correlated with soil nutrient heterogeneity. Namely, plant height variation was significantly correlated with soil total N and phosphorus heterogeneity, and chlorophyll variation was positively correlated with soil organic carbon heterogeneity.

**Figure 2:**
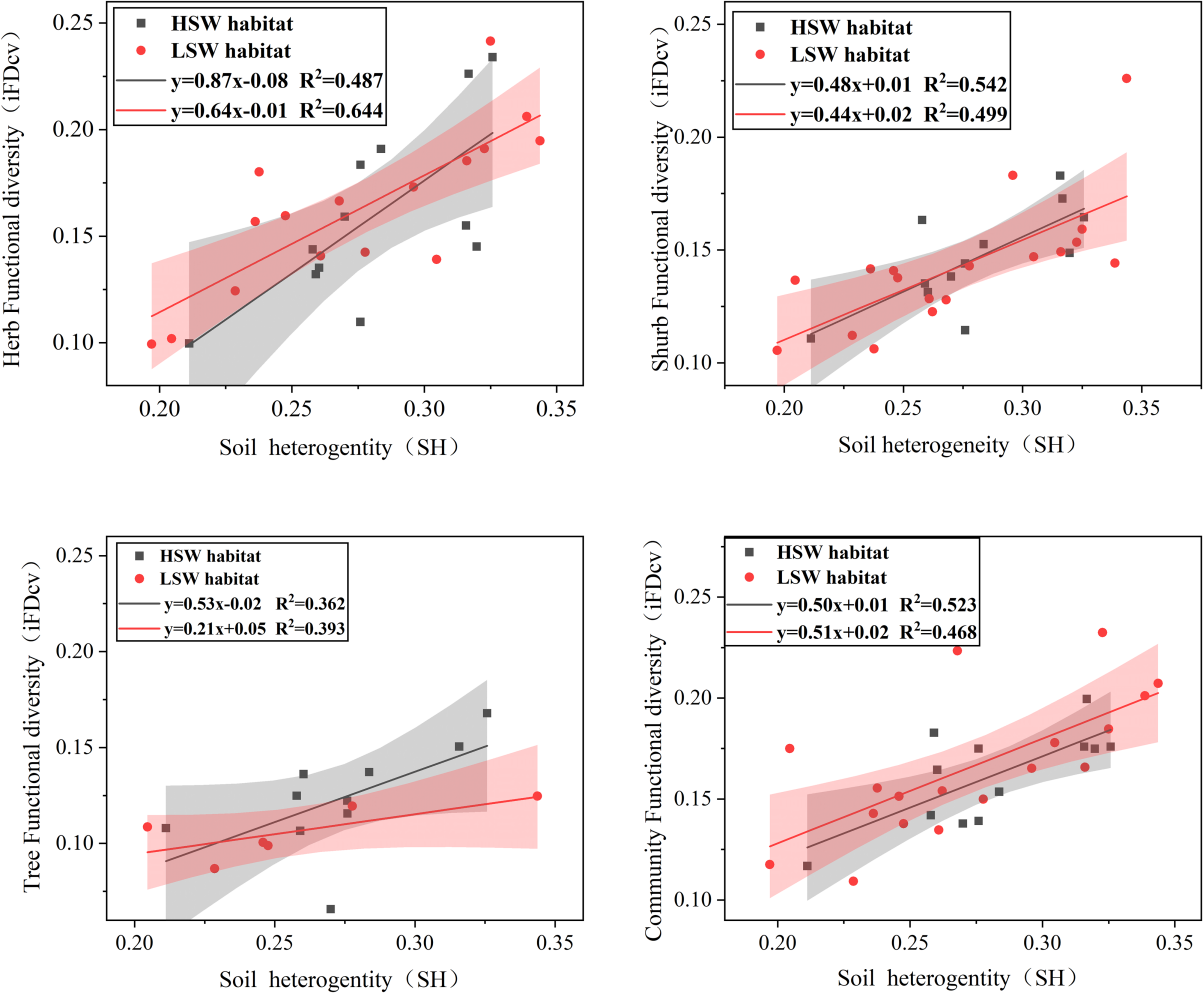
Regression analysis resulted in a significant positive correlation between intraspecific functional trait variation iFD_CV_ (trees, shrubs, herbs, and communities) and soil environmental heterogeneity. Confidence intervals (95%) were drawn. (*P<0.01)*.

The results of the linear regression modeling revealed a significant and positive correlation (*p<0.01*) between iFD_CV_ in trees, shrubs, herbs, and communities and soil heterogeneity (SH). In the HSW habitat, impacts of SH on ITV among different life forms showed a pattern of shrubs > herbs > trees. However, in the LSW habitat, the pattern shifted to herbs > shrubs > trees. Furthermore, the regression results for herbs and trees were higher in the low moisture-salinity environment, while the opposite trend was observed for shrubs and communities (**Figure 2**).

**Figure 3:**
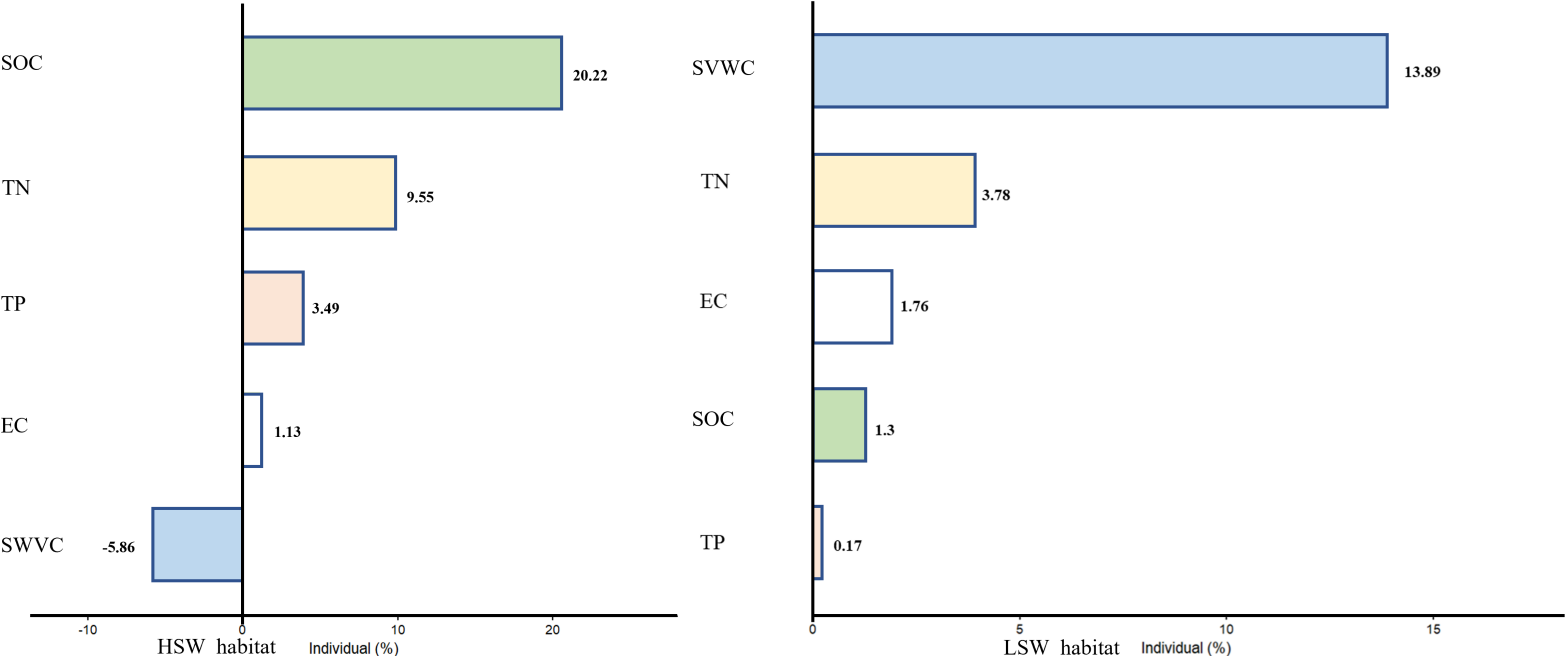
The effect of soil environmental heterogeneity on community traits is illustrated in the figure, which shows the results of variance decomposition and hierarchical partitioning, where the bars are the solitary effects of the variables and the numbers are the percentage of variance explained by the corresponding environmental factors.

The results of exemplar analysis, which aimed to illustrate the impacts of soil heterogeneity on intraspecific plant trait variation at the community level, revealed that both hierarchical partitioning and variance breakdown of soil environmental heterogeneity made distinct contributions (**Figure 3**). In HSW habitat, SOC individually accounted for up to 20.22% of the variation in intraspecific traits at the community level, followed by soil TN (9.55%), soil TP (3.49%), and soil electrical conductivity (EC) (1.13%). In the LSW habitat, soil SVWC explained 13.89% of the total variation, with soil TN (3.76%), soil EC (1.76%), soil SOC (1.3%), and soil TP (0.17%) contributing subsequently.

## 4 Discussion

A notable ecological problem lies in understanding the effects of soil environmental heterogeneity on plant intraspecific trait variation (Stark et al., 2017; Vernham et al., 2024). This study provides compelling evidence that, in the arid zone, soil heterogeneity plays a substantial role in driving intraspecific trait variation at the community level and across different plant life forms. Furthermore, the study highlights that the degree to which individual components of soil environmental heterogeneity contribute to trait variation varies depending on soil moisture and salinity regimes. These findings shed light on the crucial role of soil heterogeneity in shaping plant intraspecific trait variation, emphasizing the significance of considering soil heterogeneity when studying plant traits in ecological contexts (Vernham et al., 2024).

### 4.1 Soil heterogeneity under different soil moisture-salinity habitats

In natural ecosystems, Zhang and Li (2019) proposed that the coefficient of variation of soil resources can be used to assess the geographical heterogeneity of soil environmental indicators in sample plots (Zhang and Li, 2019). Grading based on the coefficient of variation of soil environment (Hu et al., 1999), soil variables like SVWC, EC, SOC, TN, and TP exhibit moderate variability (10% < CV < 100%), indicating heterogeneity. In HSW habitat, the presence of abundant and unevenly distributed surface salt sources leads to increased salt aggregation in the topsoil layer of various plant communities. Consequently, soil EC heterogeneity is significantly higher in HSW habitat compared to LSW habitat. On the other hand, changes in soil surface water content are more pronounced in drier conditions with reduced vegetation cover, influenced by factors such as soil type and aridity. HSW habitat exhibit higher soil nutrient heterogeneity (SOC, TN, and TP) compared to LSW habitat due to the relative conditions for vegetation growth, resulting in a greater variety of plant species and higher vegetation cover. Increased litter contributes to organic matter accumulation in the soil. Nutrients in the soil are essential for plant growth, biomass accumulation, and tissue development (Ågren et al., 2012; Kerkhoff et al., 2006). The concentration of soil phosphorus is primarily influenced by the parent material and the extent of rock weathering. Our study site, situated in the arid region of northwest China, predominantly consists of calcareous and alkaline soils, which further shape the levels of phosphorus in the soil (Rezapour, 2014), soil phosphorus exists in the form of calcium phosphate, resulting in the least heterogeneity among different soil moisture-salinity habitats.

In smaller-scale research sites such as islands, depressions, and karst sinkholes, the response of abiotic elements to plant communities is more evident (Yang et al., 2021). The greater the habitat heterogeneity, influenced by factors like topography, soil moisture, and nutrients, the better it can support the growth requirements of various specie (Ma et al., 2017; Yang et al., 2017). For instance, Shui et al (2022) found that soil total nitrogen and water content had significant effects on plant functional trait variation at the sample site scale (Shui et al., 2022). Habitat heterogeneity plays a significant role in intraspecific trait variation, as confirmed by Yu et al. (2013), who found that soil heterogeneity controlled the pattern of species distribution at small scales and reflected its impact on plant adaptation (Yu et al., 2013). Soil environmental heterogeneity expands the ecological niche space for plants and improves the regulation of community functional traits through environmental filtration, as highlighted by Liu and Ma (2015), plants often adjust their morphological, physiological, and chemical traits to mitigate the adverse effects of environmental changes (Liu and Ma, 2015). Overall, HSW habitat exhibit higher soil heterogeneity compared to LSW habitat, indicating that ITV may higher and soil heterogeneity may have a greater influence on intraspecific trait variation in HSW habitat than in LSW habitat. This analysis serves as a theoretical basis and point of reference for forecasting plant adaptation strategies in the event of environmental change.

### 4.2 Intraspecific traits variation under different soil moisture-salinity habitats

Consistent with expectations, ITV in plant traits was higher in HSW than in LSW habitat. The coefficients of variation for intraspecific plant functional traits ranged from 3.85% to 29.41% in the HSW habitat, with a mean value of 15.84%; LSW habitat are from 4.69% to 26.77%, with a mean value of 15.02% (**Table 1**). Interestingly, this is different from Zhong et al (2018) finding of variation in functional traits of woody plants in the Qianzhong Karst (Zhong et al., 2018). This discrepancy may be attributed to the fact that different species are exposed to varying forms and intensities of external biotic factors (such as species composition) and abiotic disturbances (such as moisture, salinity, and nutrient heterogeneity), leading to substantial variations in their effects on plant traits (Ding et al., 2011). Furthermore, Umaña and Zhang (2015) observed limited ITV in common species, suggesting a convergent strategy wherein plants adapt to their habitats to efficiently utilize available resources (Grime, 2006). The range of plant trait variation observed in different soil moisture-salinity habitats in our study can be explained by plants striving to strike a balance between costs and benefits. This phenomenon aligns with previous research demonstrating that species and trait composition exhibit variations along environmental gradients. Each plant’s unique approach represents an optimal trade-off for its specific environmental conditions (Reich et al., 2003). In other words, ITV confers advantages to plants by enabling them to effectively adapt to their environmental conditions.

Plants of different life forms have adapted to their growing environments, exhibit variations in resource-use efficiency as reflected in the chemical, physiological, and morphological traits of leaves. The seven species selected in this study, originating from the life forms of trees, shrubs, and herbs, revealed significant ITV in leaf traits within the same soil moisture-salinity habitat (**Table 2**), underscoring the divergence of plant nutrient use strategies during species evolution (Niu et al., 2013). Intraspecific traits variation in the six functional traits of herbs, shrubs, and trees in the two soil moisture-salinity habitats were generally exhibited a trend of herbs > shrubs > trees. This suggests that herbaceous plants are more adaptable compared to shrubs and trees. Both within communities and within life forms, ITV was often greater in high soil moisture-salinity habitat than in low soil moisture-salinity habitat. It is noteworthy that the two soil moisture-salinity habitats showed the greatest change in plant height and the least change in leaf carbon content. A previous study found that different functional traits exhibited varying levels of ITV, with plant height accounting for 69% of the total variation, offering a potential explanation for the substantial intraspecific range in plant height across all life forms (Zhu et al., 2011). The C element, constituting the cytoskeleton of plants, is generally stable and internally robust, making it less susceptible to variations induced by changes in the external environment. This is consistent with the lower coefficient of variation of leaf C at the community level in our study (**Table 1**).

**Table 2:** Changes in ITV of species in two soil moisture and salinity habitats. leaf carbon(C), leaf nitrogen (N), leaf phosphorus (P), leaf thickness(LT), plant height(H), chlorophyll(CHl), intraspecific functional trait variation (iFD_CV_).

Species Plant trait		*Populus euphratica*	*Alhagi sparsifolia*	*Apocynum venetum*	*Nitraria tangutorum*	*Halimodendron halodendron*	*Phragmites australis*	*Karelinia capsica*
C	HSW	3.85	4.32	4.80	4.77	4.13	5.27	7.01
	LSW	4.69	5.58	5.58	4.55	3.19	5.32	5.59
N	HSW	10.09	20.15	16.78	12.93	12.88	20.37	21.93
	LSW	15.58	17.62	11.37	13.11	7.70	20.59	15.02
P	HSW	6.56	16.68	13.29	11.10	13.43	17.20	18.32
	LSW	11.57	14.90	15.69	14.62	15.81	22.41	20.80
LT	HSW	11.50	11.82	17.50	10.78	6.86	14.53	19.61
	LSW	13.09	14.03	13.90	12.16	8.16	15.62	21.62
CHl	HSW	8.30	7.41	14.13	9.25	11. 24	9.41	17.75
	LSW	7.11	7.11	13.49	11.86	16.72	8.94	12.21
H	HSW	24.42	28.81	25.24	31.74	15.51	31.78	23.26
	LSW	20.40	24.92	18.91	26.69	13.68	26.19	21.55
iFD_CV_	HSW	10.79	14.87	15.29	13.43	10.68	16.43	17.98
	LSW	12.07	14.03	13.16	13.83	10.88	16.51	16.13

We observed that different habitats significantly influenced the ITV in leaf thickness. In arid environments with limited soil moisture, plants allocate more leaf photosynthetic products towards building protective tissues or increasing chloroplast density to mitigate leaf damage or water loss due to high temperatures, thereby enhancing water use efficiency (Dong et al., 2019). Leaf thickness is a particularly sensitive leaf trait for plants, as evidenced by the greater ITV in leaf thickness observed at the community level in LSW habitat compared to HSW habitat in our study. For instance, plants such as *Phragmites australis* thrive in moist habitats, explaining why herbs species dominate in HSW habitat, while shrubs like *Apocynum venetum*, with thicker leaves, are better adapted to more drier environments, leading to a predominance of shrub species in LSW habitat. This phenomenon may be attributed to the evolutionary development of ecological strategies by vegetation communities during natural succession, tailored to specific habitat conditions and resulting in more stable trait characteristics. Chlorophyll serves as a key indicator of a plant’s photosynthetic capacity, and it has been noted that drought stress can alter chlorophyll content (Guan et al., 2003). This aligns with our findings of higher ITV in chlorophyll content in LSW habitat, suggesting that the community actively adapts to arid environments by modifying chlorophyll levels. Furthermore, previous studies have indicated that shrubs exhibit greater salt tolerance than trees, and both shrubs and herbs surpass trees in terms of water holding capacity (He et al., 2006; Holdaway et al., 2011). According to these results, plant functional traits variation is somewhat influenced by life form, and ITV among different life forms reflect differing environmental constraints and adaptive strategies (Símová et al., 2018). Overall, higher ITV could potentially strengthen plants’ resilience to severe conditions and improve their adjustment to extreme environment (Niu et al., 2020), which may be a manifestation of desert plants’ response to heterogeneous environments. Therefore, disregarding ITV may underestimate the response of community-level traits to environmental change.

### 4.3 Heterogeneity of soil factors enhances intraspecific trait variation of desert plant

Our investigation uncovered that soil environmental heterogeneity had a discernible impact on ITV across life forms, following a general pattern of herb > shrub > tree. This suggests that herbs may be more susceptible to the influence of soil heterogeneity and exhibit greater tolerance and resistance compared to shrubs and trees. The strong correlation between plant height variation and soil nitrogen and phosphorus heterogeneity suggests strong effects of soil nitrogen and phosphorus distribution on plant growth (Kerkhoff et al., 2006). The results were consistent with our expectations, namely, high soil heterogeneity in HSW habitat may promotes high ITV of plant height. The ITV of leaf N and P may partly depend on the content and distribution of N and P in the soil (Berman-Frank and Dubinsky, 1999). For example, a global analysis conducted by Ordonez revealed a positive correlation between the mass fraction of nitrogen and phosphorus in leaves and the availability of these elements in the soil. Therefore, the mass fraction of leaf nitrogen showed a positive correlation with soil phosphorus levels (Ordoñez et al., 2009). In addition, leaf nitrogen is directly related to water availability, storage and resource acquisition (Dong et al., 2019; Li et al., 2020).

Our study yields two main findings: (1) soil heterogeneity improve desert plant ITV; (2) The contribution of each soil factor heterogeneity to the ITV at community-level traits varies and changes with soil moisture and salinity conditions. Consistent with our expectations, soil heterogeneity had a higher effect on ITV in herbs and shrubs than in trees, suggesting that herbs and shrubs are more sensitive to soil heterogeneity. ITV increases with soil heterogeneity (**Figure 2**), conforming to the ecological niche theory, which illustrates the connection between plant species’ environmental exposures and the diversity of their traits (Bucher et al., 2016; König et al., 2018). The ELWNNR, situated in a typical desert zone with infertile, arid soils and slow nutrient cycling, plant growth and distribution would be more obviously limited (Titus et al., 2002). At the community level, soil organic carbon heterogeneity in HSW habitat independently accounts for the highest percentage of plant functional traits variations (20.22%), followed by TN (9.55%) and TP (3.49%). Nutrient-deficient soils trigger a transition from species that are typically associated with rapid resource acquisition to those that are linked with resource preservation. This phenomenon, observed in our study, showed the adaptive response of plant communities to nutrient limitations in their environment. As nutrients become scarce, plants tend to prioritize conserving and efficiently utilizing available resources rather than investing in traits that promote rapid growth and resource acquisition (Diaz et al., 2004; Ordoñez et al., 2009). In LSW habitat, soil moisture and nitrogen heterogeneity are significant environmental factors influencing plant adaptation, with soil SVWC (13.89%) and TN (3.78%) heterogeneity explaining the highest rates of variation, respectively (Titus et al., 2002). This finding aligns with the observation that community chemical traits in typical grassland ecosystems respond to nitrogen and water enrichment and are dominated by ITV (Violle et al., 2007). Furthermore, plants in LSW habitat are more susceptible to drought stress than nutrient deficiency, as drought stress has been found to limit nutrient uptake, subsequently inhibiting photosynthesis and plant growth (Farooq et al., 2009; Jaleel et al., 2009; Pérez-Harguindeguy et al., 2016).

## 5 Conclusions

In summary, this study highlights the significant promoting influence of soil heterogeneity on ITV of desert plants in the ELWNNR, with this influence being more pronounced in herbs and shrubs, and varying with different soil moisture-salinity habitats. ITV of desert plant in the two habitats reflect both the habitat filtering effect of soil heterogeneity at small scales and the diverse adaptive mechanisms of plants to their habitats. Microhabitats provide the necessary space for these species to coexist. Consequently, soil heterogeneity plays a crucial role in enhancing trait variation in plant communities, and maximizing the survival efficiency of desert plant species in the ELWNNR. In summary, soil heterogeneity promotes multi-species coexistence in arid zones. Our findings provide a scientific foundation for conservation planning in arid land, it is essential to protect and enhance soil heterogeneity within habitats, which will serve as a buffer against extreme environmental impacts.

## Data Availability

The datasets presented in this study can be found in online repositories. The names of the repository/repositories and accession number(s) can be found in the article/**Supplementary Material**.
