# Supplementary material for: Spatial heterogeneity of soil factors enhances intraspecific variation in plant functional traits in a desert ecosystem
Source: Front Plant Sci. 2024 Dec 23;15:1504238. doi: 10.3389/fpls.2024.1504238 (PMC11721652; doi:10.3389/fpls.2024.1504238)
Supplement: Supplementary file 1 [file Supplementaryfile1.docx]

***Supplementary Material***

**Table S1** Seven plant species information.

| **Plant species name** | **Family** | **genera** | **life form** | **abundance** | |
| --- | --- | --- | --- | --- | --- |
|  |  |  |  | **High** | **Low** |
| *Phragmites australis* | *Fabaceae* | *Phragmites* | **Herb** | 2728 | 2113 |
| *Karelinia capsica* | *Amaranthaceae* | *Suaeda* |  | 257 | 228 |
| *Nitraria tangutorum* | *Zygophyllaceae* | *Nitraria L* | **Shurb** | 119 | 200 |
| *Halimodendron halodendron* | *Fabaceae* | *Halimodendron Fisch* |  | 115 | 17 |
| *Apocynum venetum* | *Apocynaceae* | *Apocynum L* |  | 1240 | 1192 |
| *Alhagi sparsifolia* | *Fabaceae* | *Alhagi Gagneb* |  | 256 | 635 |
| *Populus euphratica* | *Salicaceae* | *Populus* | **Tree** | 66 | 25 |

**Figure S1:** Geographic locations of sites at different strips in the study area. Strips were set up at the northern bank of the Aqikesu River (c)in the ELWNNR(b), located in the southwest of the Junggar Basin, Xinjiang (a). Three sample strips spaced 1 km apart and running perpendicular to the river channel (c). Within each strips, 10 sample sites were established at intervals of 0.1 km, at each sample site, three 10 m × 10 m plots were investigated, with a spacing of about 100 m between plots(c). To ensure clarity, the spacing between strips, sites and plots in the figures has been slightly enlarged.


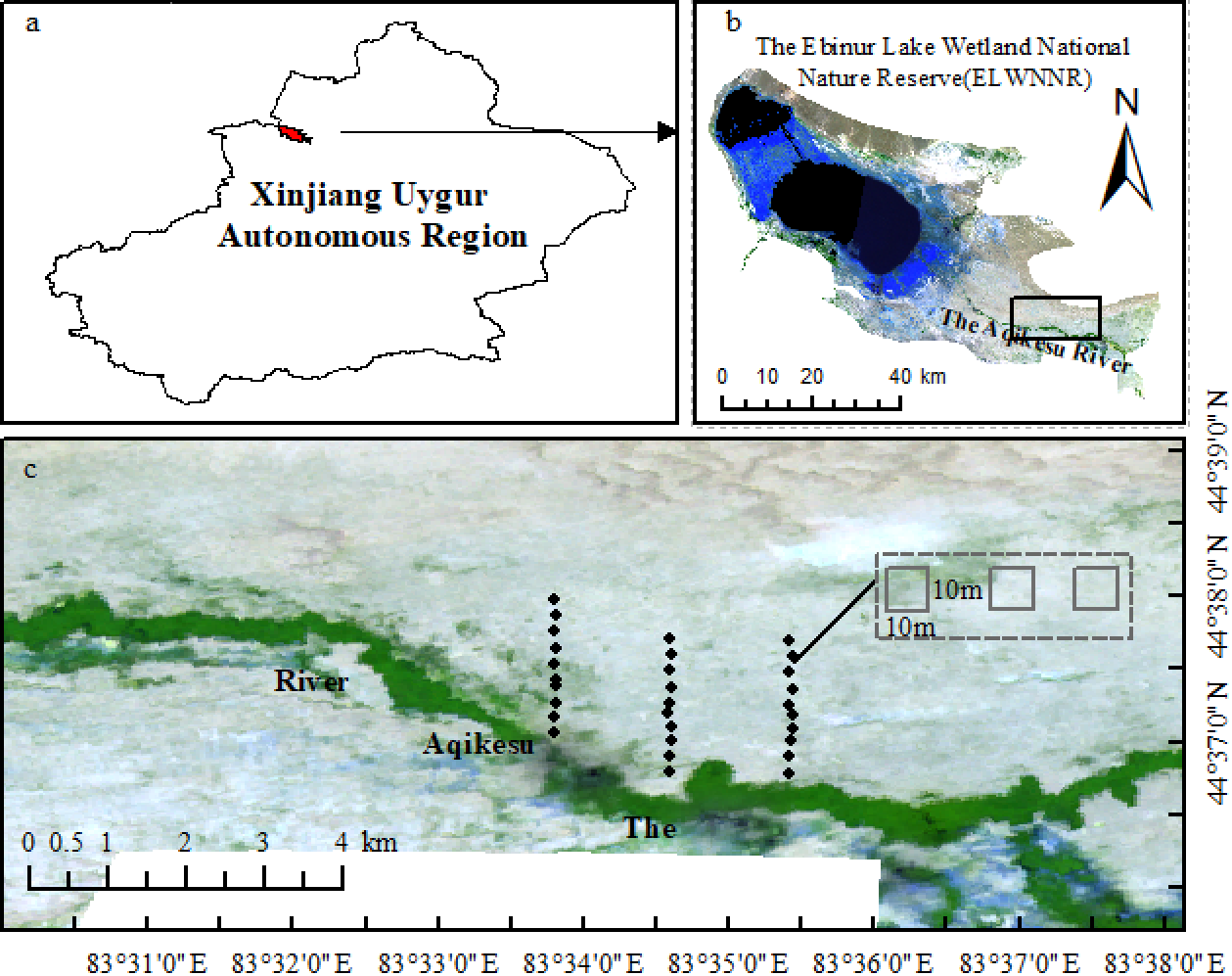


**Figure S2:** A visualized correlation matrix was developed based on the Pearson correlation coefficients of community trait variability (CWV trait) and soil environmental contextual factors (CV factor) in the Ebinur Lake Wetland National Nature Reserve (ELWNNR)). The size of the circle reflects the correlation coefficient; the higher the correlation coefficient (in both positive and negative directions), the larger the circle. Significance levels: **=P <0.01, *=P <0.05.
